# A Radiation-Free Classification Pipeline for Craniosynostosis Using Statistical Shape Modeling

**DOI:** 10.3390/diagnostics12071516

**Published:** 2022-06-21

**Authors:** Matthias Schaufelberger, Reinald Kühle, Andreas Wachter, Frederic Weichel, Niclas Hagen, Friedemann Ringwald, Urs Eisenmann, Jürgen Hoffmann, Michael Engel, Christian Freudlsperger, Werner Nahm

**Affiliations:** 1 Institute of Biomedical Engineering (IBT), Karlsruhe Institute of Technology (KIT), Kaiserstr. 12, 76131 Karlsruhe, Germany; andreas.wachter@kit.edu (A.W.); werner.nahm@kit.edu (W.N.); 2 Department of Oral, Dental and Maxillofacial Diseases, Heidelberg University Hospital, Im Neuenheimer Feld 400, 69120 Heidelberg, Germany; reinald.kuehle@med.uni-heidelberg.de (R.K.); frederic.weichel@med.uni-heidelberg.de (F.W.); juergen.hoffman@med.uni-heidelberg.de (J.H.); michael.engel@med.uni-heidelberg.de (M.E.); christian.freudlsperger@med.uni-heidelberg.de (C.F.); 3 Institute of Medical Informatics, Heidelberg University Hospital, Im Neuenheimer Feld 130.3, 69120 Heidelberg, Germany; niclas.hagen@med.uni-heidelberg.de (N.H.); friedemann.ringwald@med.uni-heidelberg.de (F.R.); urs.eisenmann@med.uni-heidelberg.de (U.E.)

**Keywords:** classification, craniosynostosis, statistical shape model, template morphing, machine learning, stereophotogrammetry, shape analysis

## Abstract

Background: Craniosynostosis is a condition caused by the premature fusion of skull sutures, leading to irregular growth patterns of the head. Three-dimensional photogrammetry is a radiation-free alternative to the diagnosis using computed tomography. While statistical shape models have been proposed to quantify head shape, no shape-model-based classification approach has been presented yet. Methods: We present a classification pipeline that enables an automated diagnosis of three types of craniosynostosis. The pipeline is based on a statistical shape model built from photogrammetric surface scans. We made the model and pathology-specific submodels publicly available, making it the first publicly available craniosynostosis-related head model, as well as the first focusing on infants younger than 1.5 years. To the best of our knowledge, we performed the largest classification study for craniosynostosis to date. Results: Our classification approach yields an accuracy of 97.8 %, comparable to other state-of-the-art methods using both computed tomography scans and stereophotogrammetry. Regarding the statistical shape model, we demonstrate that our model performs similar to other statistical shape models of the human head. Conclusion: We present a state-of-the-art shape-model-based classification approach for a radiation-free diagnosis of craniosynostosis. Our publicly available shape model enables the assessment of craniosynostosis on realistic and synthetic data.

## 1. Introduction

### 1.1. Craniosynostosis

Craniosynostosis is characterized by the premature fusion of skull sutures in infants and results in irregular growth patterns. The reported prevalence is three to six cases per 10,000 live births [1,2,3]. Craniosynostosis can occur isolated (affecting one suture) or non-isolated (affecting multiple sutures). Syndromic conditions such as Crouzon, Muenke, or Pfeiffer syndromes have genetic reasons and lead to multi-suture synostosis. These syndromes tend do show phenotypical craniofacial findings. Unlike syndromic cases, the causes of isolated craniosynostosis are believed to be multifactorial. Hereditary conditions and genetic mutations have been identified to cause premature fusion of specific sutures [4]. Symptoms of isolated craniosynostosis are a deformity of the neurocranium and, consecutively, viscerocranium. Craniosynostosis has been linked to elevated intracranial pressure [5], which can lead to reduced brain growth and reduced neuropsychological development [6]. Depending on the involved suture, isolated craniosynostosis can be classified into sagittal synostosis (scaphocephaly), metopic synostosis (trigonocephaly), unilateral coronary synostosis (anterior plagiocephaly), lambda synostosis (posterior plagiocephaly), and bicoronal synostosis (brachycephaly). Although brachycephaly includes the synostosis of both coronal sutures, the medical community counts it among isolated synostosis. Surgical treatment involves resection of the synostosis, as well as remodeling and reshaping of the cranial vault. The operation aims to prevent abnormal brain growth, thus enabling a regular development of the skull and face [7,8]. Complications during surgery are rare [9], and in most cases, a normalized head shape is achieved [10]. The most important differential diagnosis for craniosynostosis are head deformities caused by positioning without suture fusion. These head deformities are mainly manifested as a non-synostotic posterior plagiocephaly. Positioning deformities are often treated with positioning pillows, helmet therapy, or changes in positioning behavior [11]. For further reading, the reader is referred to [12].

As determined by Virchow’s Law, the premature closure of a suture limits the expansion of the skull perpendicular to the fused suture and causes compensatory growth along the suture, resulting in distinct head shapes [13]. During diagnosis, physicians perform visual examination, palpation, cephalometric measurements, and medical imaging. ct imaging is the gold standard for diagnosis, as well as for surgical planning and is routinely performed in many craniofacial centers worldwide. However, this exposes infants to ionizing radiation, which should be avoided [8]. One alternative imaging method is Black Bone mri [14,15], which has the notable drawback that the infant needs to be sedated during image acquisition to prevent child from moving. Sonographic examinations [16] and 3D photogrammetry are radiation-free and broadly available diagnostic options. Photogrammetric scans provide inexpensive and fast means to objectively quantify head shape without exposure to radiation or sedation. They are often used to monitor the condition before surgery and the head development after the operation [17]. For more information about the current development, we refer to [16].

### 1.2. Assessment and Classification of Craniosynostosis Using Statistical Shape Modeling

Statistical shape modeling describes the approach to capture variations of geometric shapes by statistical methods. pdm are the most common type of ssm and use a point cloud representation. Cootes et al. [18] introduced the idea to construct pdm using training instances. pdm require a registration step and correspondence among training instances before computing the statistical properties of the training data. Usually, pca is applied to determine the principal components according to their respective variance in the training data. Synthetic, valid shapes are constructed by linear combinations in the vector space defined by the principal components and constrained by their respective eigenvalues. The idea to model statistical attributes has been introduced in facial models [19,20], while the Liverpool-York-Head-Model is the first publicly available shape model of the full human head in both shape and texture [21], also including the first publicly available child model constructed from healthy subjects between 2 and 15 years. Egger et al. [22] gives a overview about face models, current trends, applications, and challenges.

SSms have also been applied to the analysis of craniosynostosis-specific head deformities. Some studies showed statistical differences between healthy subjects and pathological subjects using pca and statistical shape modeling on photogrammetric surface scans [23], while other studies built healthy models and found shape vector differences between pre- and post-operative craniosynostosis patients [21,24,25].

While for ct data, ssm have been successfully used to classify craniosynostosis in combination with other features [26], no ssm-based classification approach has been presented for 3D photogrammetric surface scans. Ray-based classification approaches have been presented [27], which do not have many of the desirable features of ssm such as a visual representation of the patient for patient counseling or the ability to create synthetic data from their training data.

### 1.3. Scope of This Work

In this publication, we construct a classification pipeline for craniosynostosis built on top of an ssm of 3D surface scans, which we also make publicly available. In particular, we make the following contributions:

- We present an alternative classification approach for craniosynostosis to distinguish between controls and three different types of craniosynostosis directly on the parameter vector of our ssm built from 3D photogrammetric surface scans. We test five different machine-learning-based classifiers on our database consisting of 367 subjects and achieve state-of-the-art results. To the best of our knowledge, we conducted the largest classification study of craniosynostosis to date.
- We propose the first publicly available ssm of craniosynostosis patients using 3D surface scans, including pathology-specific submodels, texture, and 100 synthetic instances of each class. It is the first publicly available model of children younger than 1.5 years and ssm of craniosynostosis patients including both full head and texture. Our model is compatible with the Liverpool-York head model [24], as it makes use of the same point identifiers for correspondence establishment. This enables combining the texture and shape of both models.
- We demonstrate two applications of our ssm, which can easily be performed with the publicly available model: First, with regard to patient counseling, we apply attribute regression as proposed by [19] to remove the scaphocephaly head shape of a patient. Second, for pathology specific data augmentation, we use a generalized eigenvalue problem to define fixed points on the cranium and maximize changes on face and ears as proposed by [28]. To the best of our knowledge, neither of these applications have been applied to patients using a craniosynostosis shape model before.

## 2. Materials and Methods

**Figure 1:**
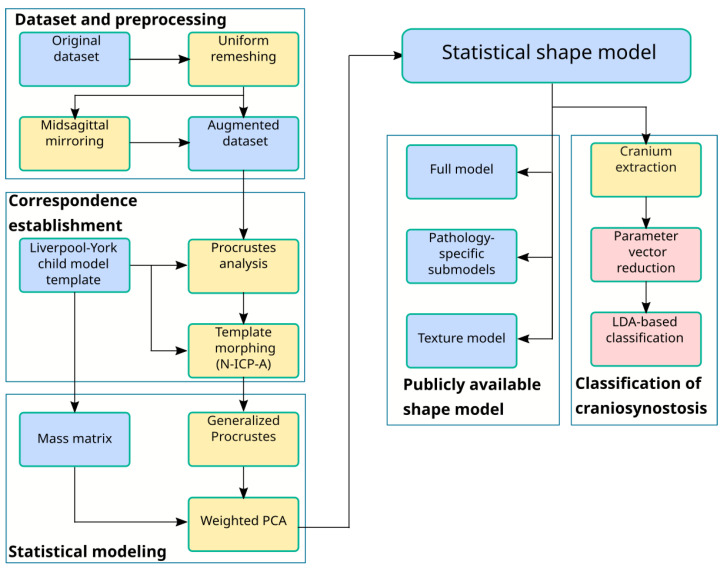
Shape model creation and classification pipeline. Blue: data, yellow: statistical and preprocessing methods, red: classification. Each of the top-level blocks is described individually.

Figure 1 gives a full overview of the pipeline from the raw data to the ssm creation and the craniosynostosis classification. We describe each of the top-level blocks in detail in the following subsections.

### 2.1. Dataset and Preprocessing

At the Department of Oral, and Maxillofacial Surgery of the Heidelberg University Hospital, photogrammetric surface scans are routinely used to monitor and document patients with craniofacial diseases. Out of the scans that were acquired between 2011 and 2020, 367 preoperative 3D photogrammetric scans were extracted. We used a standardized protocol, which had been examined and approved by the Ethics Committee Medical Faculty of the University of Heidelberg (Ethics Number S-237/2009). The study was carried out according to the Declaration of Helsinki, and written informed consent was obtained from parents. The Canfield VECTRA-360-nine-pod system (Canfield Science, Fairfield, NJ, USA) was used for recording. To avoid artifacts on the head due to hair, the scanned infants wore tight-fitting nylon caps. For each of the recordings, the dataset provided the 3D vertex coordinates, UV texture coordinates, and the triangular face indices connecting the vertices to a mesh surface. Each recording contained additional metadata, which includes the medical diagnosis of the physician, the patient’s age on the day of the recording, and 10 cranial and facial landmarks manually annotated by a medical expert. We summarize the aforementioned landmarks in Table S1.

**Figure 2:**
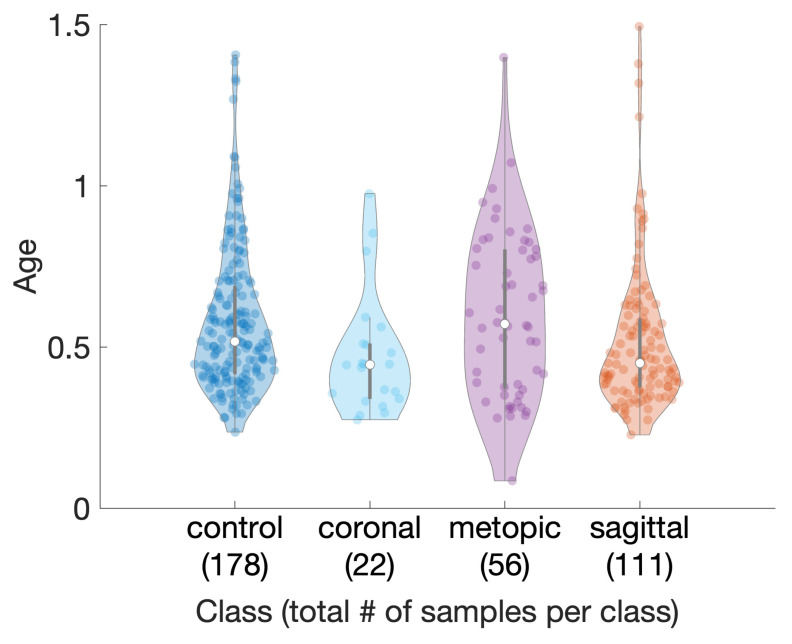
Age distribution among classes of the dataset. Parenthesis indicate number of samples per class.

We retrieved patient scans classified with one of three types of craniosynostosis, namely “coronal suture fusion” (brachycephaly and unilateral anterior plagiocephaly), “sagittal suture fusion” (scaphocephaly), “metopic suture fusion” (trigonocephaly), as well as a control group without any suture fusion. This makes our approach comparable to other classification studies, which distinguished between craniosynostosis and non-craniosynostosis classes, in particular [26,27]. Besides healthy subjects, our control group contained also scans of children with positional posterior plagiocephaly without suture fusion, who were later treated with helmet therapy or laying repositioning. All craniosynostosis patients later underwent surgical remodeling of the cranium. We show violin plots [29] using a publicly available implementation [30] of the subjects’ age distribution in Figure 2.

During recording, the patients had to be held tight; often, the neck of the patient was covered by clothes or the hands of the medical staff. For this reason, some recordings contained isolated parts and other artifacts. Additionally, close to the ears, we often found mesh irregularities such as large edge lengths. The open-source tool *Meshlab* (ISTI-CNR, Pisa, Italy) [31] was used to remove isolated parts and duplicated vertices and to close holes. After artifacts removal, we used isotropic explicit remeshing [32] to avoid large edge lengths and to obtain a regular, uniform surface scan.

Depending on the model application, it can be advantageous to use a non-regular mesh (having different resolutions on different parts of the mesh). Cranial parts mostly change smoothly in comparison with facial parts and can thus be expressed with fewer vertices and lower spacial resolution to reduce computational cost.

We used the mean shape of the Liverpool-York child head model [21] as a template for correspondence establishment. This template has the advantage of being symmetric and does not have any head deformity, avoiding pathological bias during model creation. To directly include eyes and close the mouth in the model, we added additional vertices into the template, which enables using the model without an additional eye model. The modified template is compatible with the original model as the order of the vertex identifiers is the same. The final template has p=13151 vertices and a mean edge length of 2.91 mm.

### 2.2. Correspondence Establishment

Correspondence establishment is crucial and has a high influence on the model performance, but is at the same time difficult to evaluate as no ground truth is available. During correspondence establishment, we intended to find corresponding points with the same morphological meaning on the template X$\in R^{p\times 3}$ and the target scans. To increase our sample size and avoid a symmetry bias for asymmetric pathological cases for the coronal model, we mirrored each subject on the midsagittal axis, thus increasing the dataset size from N=367 to 2N=734. To prepare template morphing, we initially aligned the template to each target using the annotated landmarks. We used Procrustes analysis to obtain a linear transformation consisting of translation, rotation, and isotropic scaling, which transformed the original template landmarks onto the corresponding target landmarks. By applying the transformation to the whole set of the template points, we aligned the template mesh to each target scan, ensuring that the facial and cranial regions of the template were close to their morphological counterparts on the target scan. Note that this process only facilitates template morphing and does neither rescale nor change the target scans.

We used nonrigid iterative closest points affine (N-ICP-A) from the family of optimal step nonrigid iterative closest points (OS-N-ICP) methods for correspondences establishment over other 3D surface registration methods that have been applied on medical data [20,21,24,33,34,35,36], as it was developed for head shape registration.

The OS-N-ICP methods were presented by [37], who based their work mostly on [35]. The core idea is to use an affine transformation for each point and locally regularize transformations of connected points. A stiffness term penalizes differences between transformations between adjacent nodes. A distance term controls how close the template vertices are transformed to the target points, and a landmark term requires that the landmark points of template and target match each other. All three terms, stiffness term, distance term, and landmark term, are optimized simultaneously using an iterative approach starting with a high stiffness. For each stiffness, a correspondence search is performed, and the optimal deformation with respect to the found correspondences is computed. As soon as the transformation changes very little, the stiffness parameter is decreased and repeated for the reduced stiffness until convergence. For detailed explanations, the reader is referred to [37]. To show that our classification approach also performs well on other registration methods, we also performed template morphing for the competing Laplace–Beltrami regularized projection (LBRP) methods. Those parts are not included in the main study and are instead available as Supplementary Material. We include mathematical descriptions in Section S2, the used hyperparameters in Table S2, and the evaluation of all the morphing methods in Section S3.

### 2.3. Statistical Modeling

A statistical analysis was performed by computing the sample mean and sample covariance matrix of the training data. To align the morphed templates, we employed rigid gpa [18]. gpa iteratively calculates the mean shape of the training data and the deviation of the training data to the calculated mean shape and aligns the training data accordingly. The Euclidean distance was used as the Procrustes distance metric. This removed the non-shape-related attributes translation and rotation from the morphed templates. For this study, we considered scale an attribute of shape because craniosynostosis-related features may depend on the patient’s age and head size. After reshaping each of our morphed templates $X_{i}\in R^{p\times 3}$ into column vectors $x_{i}\in R^{3p}$, we stacked them horizontally to obtain the observation matrix $X_{\mathrm{Obs}}\in R^{3p\times 2N}$. We regarded them as independent observations, which served as training data upon which we built our ssm. We computed the mean shape $\bar{x}\in R^{3p}$, and by subtracting the mean shape from the observations matrix, we obtained the mean-aligned data matrix, which we refer to as zero-mean data matrix:

(1) $$X_{\mathrm{ObsZM}}=X_{\mathrm{Obs}}-\bar{x}$$

To compute the eigenvectors and eigenvalues of the sample covariance matrix of the zero-mean training data, we used weighted principal component analysis (WPCA). In contrast to ordinary pca (which would over-emphasize regions with high point densities such as the face), WPCA enables all parts of the head to have the same influence on the principal components.

We defined the mass matrix $M\in R^{p\times p}$, composed of per-vertex weights and per-edge weights, in a very similar manner to barycentric cells. The diagonal elements of M represent the vertex weights. Each vertex weight is defined as the sum of the area of the adjacent faces for which this vertex is the nearest neighbor. Likewise, the non-diagonal elements represent the edge weights, and each edge weight is defined as the sum of the area of the adjacent faces for which this edge is the closest edge. To account for the vectorized representation of the observations, the mass matrix is then stretched by a factor of 3 and nearest-neighbor-interpolated, resulting in $M_{3}\in R^{3p\times 3p}$. We computed the weighted Gram matrix $G_{W}\in R^{2N\times 2N}$ as

(2) $$G_{W}=X_{\mathrm{ObsZM}}^{T}M_{3}X_{\mathrm{ObsZM}}$$

and performed an eigendecomposition of $G_{W}$ using

(3) $$G_{W}=U_{G}\Lambda_{G}U_{G}^{T}.$$

We computed the principal components $V\in R^{3p\times 2N}$ of the training data as

(4) $$V=X_{\mathrm{ObsZM}}U_{G}\Lambda_{G}^{-\frac{1}{2}},$$

and the eigenvalues $\Lambda \in R^{2N\times 2N}$ of the sample covariance matrix of the training data by re-scaling the eigenvalues of the Gram matrix:

(5) $$\Lambda =\frac{1}{2N-1}\Lambda_{G}.$$

Each observation could then be defined using the principal components and eigenvalues using

(6) $$x=\bar{x}+V\Lambda^{\frac{1}{2}}\alpha .$$

As the data matrix was centered before, the last eigenvalue will be zero and can be omitted.

We created one full model, four class-specific submodels, and one cranium-only model. The full model was created using the full zero-mean observation matrix. The class-specific submodels used the assigned diagnosis label for each observation. For the classification approach, we built a cranial model and extracted the cranial part of the template to remove possible influences of the face. For each model, gpa was performed individually.

### 2.4. Classification of Craniosynostosis

The classifier was trained on the cranium model to distinguish between the labeled classes and, thus, between the three different types of craniosynostosis and the control class. We extracted the coefficient vector α for each observation from the cranial model, which served as an input for the classifiers. Using the coefficient vector as shape descriptors and as a direct input for an svm has been successfully tested in a different domain [38].

We evaluated five different machine-learning-based classifiers: svm [39], lda [40], nb [41], bdt [42], and knn [43]. All classifiers were implemented using the Python module *scikit-learn* [44] (Version 1.0.2), mostly sticking to the default settings. svm are binary classifiers that use kernel functions to map the input parameters into a high-dimensional representation, which can be separated by hyperplanes. We chose a kernel-based on radial basis functions and a multi-class model with 6 one-versus-one binary classifiers. For lda, we used a multivariate Gaussian distribution for each class assuming the same covariance matrix for each class. Each prediction is assigned to the class whose mean is the closest in terms of the Mahalanobis distance taking into account the prior probability of each class. nb assumes conditional independence between input variables. Similar to lda, we used a Gaussian model to distinguish between classes. knn classification classifies the test sample according to the *k* closest neighbors. We selected k=5 nearest neighbors in Euclidean space. For tie-breaking we chose the nearest neighbor among the tied classes. bdt are white-box classification algorithms using a hierarchical, tree-like structure. We used the default implementation for tuning the hyperparameters of the bdt.

We used stratified 10-fold cross-validation on the unmirrored samples. For each split, the test set was only composed of the original, unmirrored samples, and the training set was augmented with the mirrored training samples. This way, each of the samples from the original set was used once for testing without the possibility of cross-over.

We ordered the principal components according to their variance, so the first principal components describe the overall shape, while the last components contain mostly noise. The noise can arise, e.g., from incorrect morphing, limited resolution, or acquisition errors during scanning. We aimed to reduce the number of principal components based on the assumption that the parameters responsible for a good classification are concentrated in the first components. We iterated over the first 100 principal components and used the accuracy as a fitness function to select the optimal number of principal components. Finally, four different metrics evaluated the final classifier: besides overall accuracy, we used g-mean, per-class sensitivity, and per-class specificity.

## 3. Results

### 3.1. Classification Results

**Figure 3:**
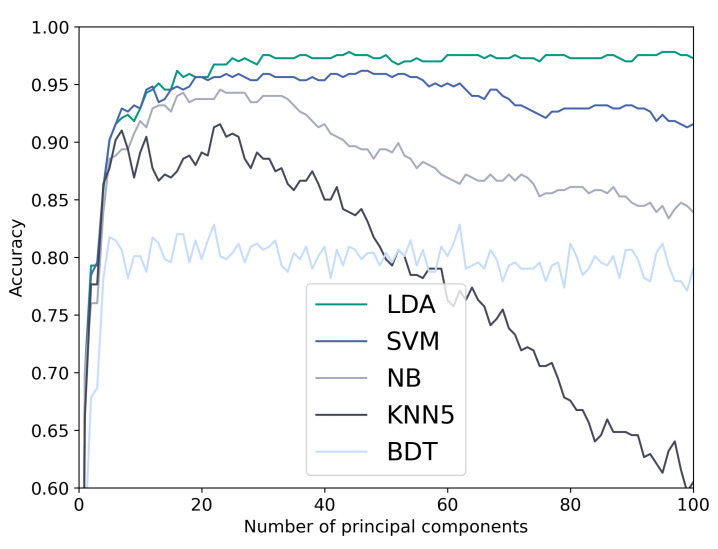
Confusion matrix, sensitivity, and specificity for lda, nonrigid iterative closest points affine (N-ICP-A) and the optimal number of components (44) using stratified 10-fold cross-validation. Con = control, Cor = coronal, Sag = sagittal, Met = metopic.

We tested the five classifiers on the cranial model. Figure 3 shows the accuracy over the used number of principal components on the N-ICP-A approach. LDA, SVM, and NB outperformed KNN and BDT. A reduction in accuracy with adding more principal components could be observed for NB and KNN and less pronounced for the SVM. For the optimal classification setup (LDA with 44 components), we show the confusion

For the optimal classification setup (LDA with 44 components), we show the confusion matrix, per-class sensitivities, per-class specificities, and g-mean in Table 1. The classifier yielded optimal per-class specificities for the pathological cases and the per-class sensitivity for the controls and metopic cases. The remaining per-class sensitivities for the pathologicalcases were between 0.773 and 0.973, while the per-class specificity for the control group was 0.958. The g-mean resulted in 0.931 and the total accuracy in 0.978. In Table S4, we include the classification results for the other morphing methods.

### 3.2. Morphing and Shape Model Evaluation

We evaluated each template morphing approach using three metrics: landmark errors, vertex-to-nearest-neighbor distances, and per-class surface normal deviations. Landmark errors provide sparse point-to-point errors on known correspondences. Vertex-to-nearest-neighbor distances evaluate how close the template has been morphed onto the target without taking into account whether the nearest neighbor is morphologically correct. What we refer to as “surface normal deviations” was proposed in [37]: we removed translational and rotational components from the morphed templates and computed surface normal deviations between the morphed templates. This evaluates how well point identifiers have been morphed onto morphologically similar regions across all scans. However, our dataset contained different pathologies, so we also expected shape and surface normal differences among different pathology classes. Hence, we modified this approach and computed surface normal deviations on each pathology class separately before calculating the cumulative mean surface normal deviations. Table 2 shows morphing errors for the N-ICP-A method.

**Figure 4:**
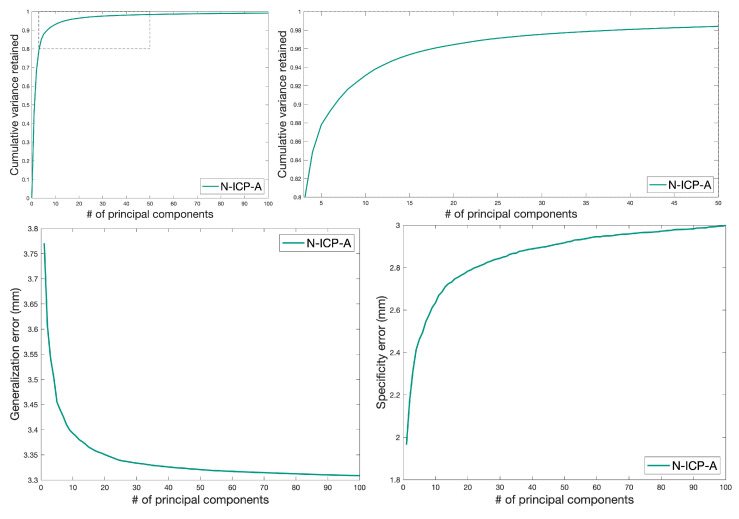
Compactness, generalization, and specificity of the final model as a function of the number of principal components. **Top left**: Compactness. **Top right**: Zoom-In. Higher is better. **Bottom left**: Generalization error. **Bottom right**: Specificity error. Lower is better.

For shape model evaluation, we used the three metrics compactness, generalization, and specificity [45,46]. Compactness determines the model’s ability to capture most of the variance with few components, generalization the model’s ability to fit to unknown observations, and specificity the model’s ability to create synthetic instances similar to the training data. We show the results for N-ICP-A in Figure 4. For the other morphing methods, we refer to Table S3.

**Figure 5:**
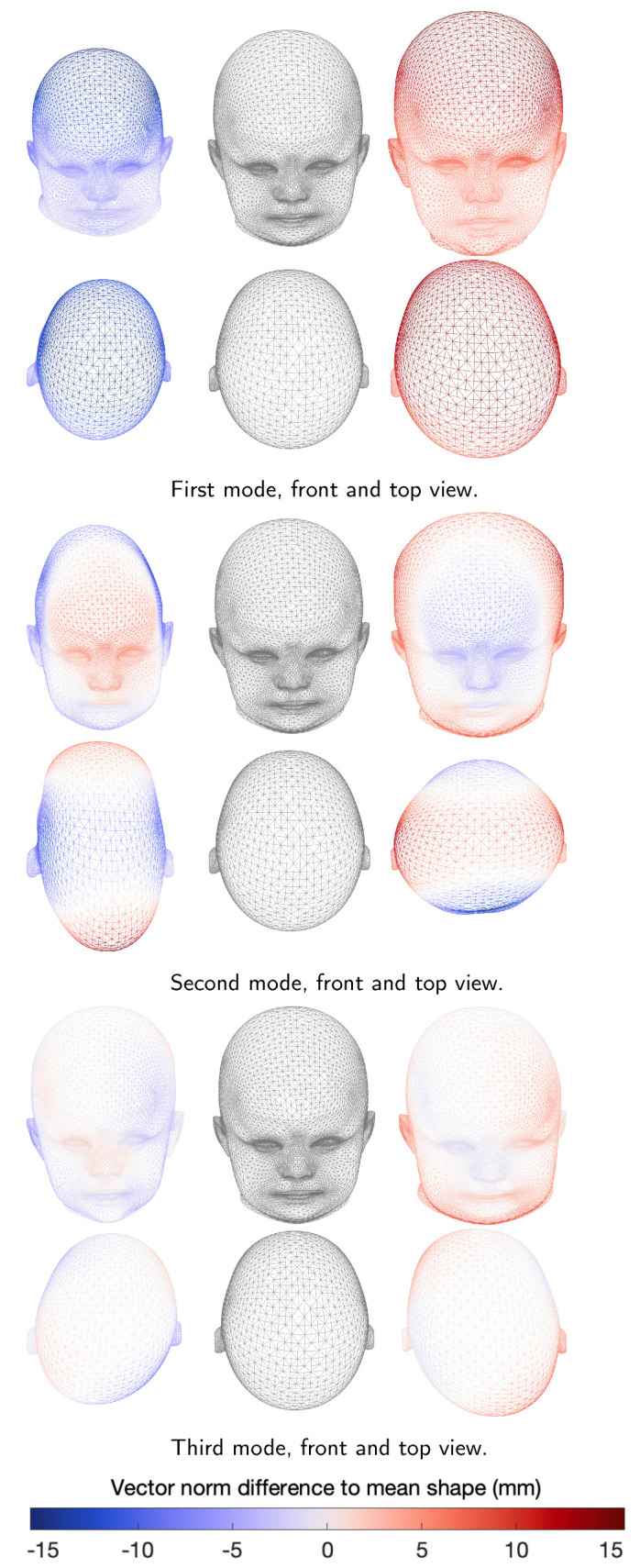
First three modes of the full model in front and top view. From left to right: −3σ, mean shape, and +3σ. Color bar indicates vector norm difference between principal component shape and mean shape (gray).

We performed a qualitative evaluation of the shape model eigenmodes and submodel mean shapes on the N-ICP-A model, as shown in Figure 5. A change of the first eigenmode of the full ssm represents a change primarily in size. For the second mode, we observed an elongated head shape characteristic of sagittal suture fusion. In the positive direction, we observed a triangular forehead typically associated with metopic suture fusion. The third mode showed head asymmetry resembling left and right plagiocephaly patients present in the control group.

**Figure 6:**
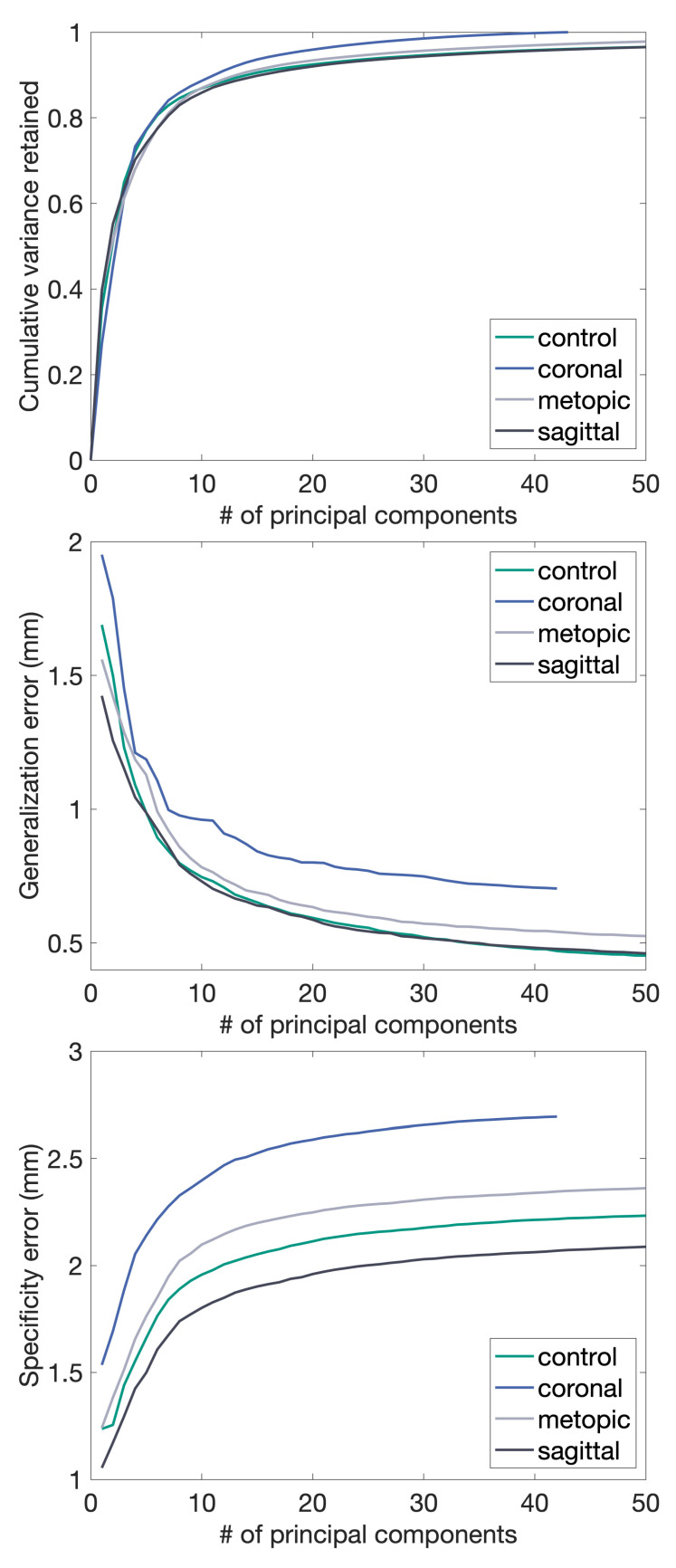
Shape model metrics for the control submodel and the pathology-specific submodels. From top to bottom: compactness, generalization, and specificity.

**Figure 7:**
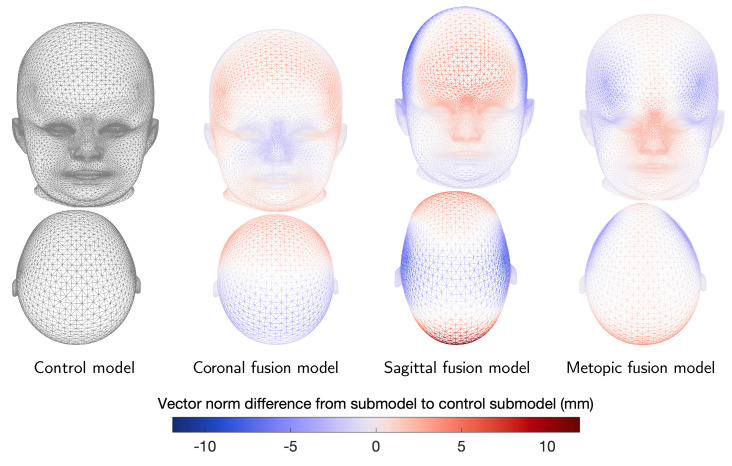
Mean shapes of pathology-specific submodels, front and top view. From left to right: control model, coronal suture fusion model, sagittal suture fusion model, and metopic suture fusion model. Color bar indicates vector norm difference between principal component shape and mean shape (gray).

In Figure 6, the resulting submodels are evaluated in terms of compactness, generalization, and specificity. However, quantitative comparisons between the submodels are invalid as the they differ in sample size. Visually, the mean shapes of each pathological submodel show the typical characteristics of each disease (Figure 7).

### 3.3. Publicly Available Shape Model

We provide the N-ICP-A-based ssm, a texture model, triangular mesh mappings, the class-specific submodels, and 100 instances of each model sampled from a Gaussian distribution. The models are publicly available online [47] under the Creative Commons license CC-BY-NC 4.0. For the models, we included 95–98% of the total variance. As we used the Liverpool-York child head model as a basis for the initial template for correspondence establishment, the statistical information of both the shape and texture of the models can easily be combined. Table 3 provides information on how many components we included in each model.

### 3.4. Shape Model Applications

We illustrate two applications of our ssm. First, we changed the head of a scaphocephaly patient toward the control group. Blanz et al. [19] presented an approach to change an attribute (such as gender or weight) using linear regression. As the pathologies in our model can be interpreted as such attributes, we changed the pathology of our samples:

(7) $$\alpha_{\mathrm{ID}},\mathrm{control}=\alpha_{\mathrm{ID}}+\alpha_{\mu ,\mathrm{control}}-\alpha_{\mu ,\mathrm{sagittal}}$$

**Figure 8:**
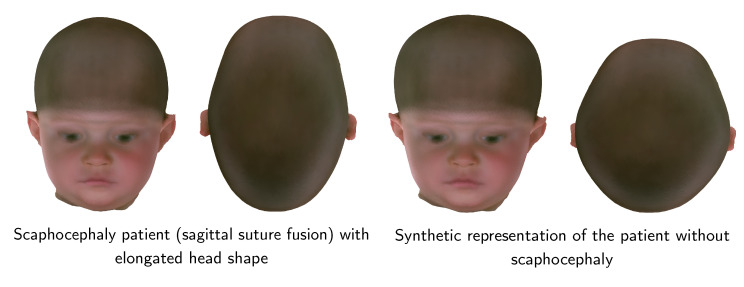
Patient pathology assessment using pathology change. Left: the original head shape of the scaphocephaly patient. Right: the patient’s head with removed pathology using our full ssm.

We present the resulting pathology change in Figure 8. This approach can be useful in clinical settings for patient counseling.

**Figure 9:**
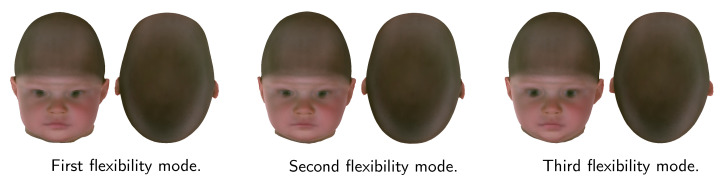
The first three flexibility modes with fixed cranium, applied to a synthetic scaphocephaly patient. Changes are minimal for the cranium and maximal for the face, neck, and ears.

Second, we sampled random instances from our shape model while keeping the points on the cranium fixed. Modeling the remaining flexibility on a shape that is held partially fixed can be described as a constrained generalized eigenvalue problem [28]. This approach can be applied to create a synthetic database for machine learning applications and is depicted in Figure 9. Alternatively, synthetic samples with a predefined pathology can also be created using a psm [48] or by simply using the pathology-specific submodel.

## 4. Discussion

We proposed the first classification pipeline for craniosynostosis based on a ssm. To the best of our knowledge, we tested it on the largest dataset used for a craniosynostosis-related classification study to date. Multiple authors [23,49] have shown that shape modeling enables a quantitative analysis of the head shape with respect to craniosynostosis. In our work, we demonstrated that ssm can not only quantify, but also classify head deformities. With an accuracy of 97.8% on 367 subjects, our approach classifies craniosynostosis comparable to competing methods: Mendoza et al. [26] achieved a classification accuracy of 95.7% on 141 subjects using ct data, and de Jong et al. [27] obtained an accuracy of 99.5% on 196 samples using a feed-forward neural network in combination with ray casting and stereophotographs. As each classification approach used different datasets, quantitative comparisons between different approaches are arguably dataset dependent. However, as we tested multiple classifiers and multiple morphing methods, we demonstrate that our classification approach is robust and does not rely on heavy hyperparameter tuning. Morphing methods for the shape model creation showed little influence in the final classification accuracy. The choice of the classifier had a larger influence on the classification results: lda and svm appeared to be the most robust classifiers with respect to the noisy components, while nb worked well with fewer than 40 components. Overall, lda worked best. In future work, a multi-class classification approach could enable a multi-suture craniosynostosis classification.

We further present the first publicly available craniosynostosis ssm. It unites statistical information of 367 subjects and their mirrored twins with and without craniosynostosis. To date, many methods presented by various authors rely on in-house datasets, making quantitative comparisons difficult. A set of synthetic photogrammetric head scans of our ssm can help in creating a large patient cohort for a reproducible evaluation of methods to assess craniosynostosis.

Our model reflects the pathologies available in our dataset: The first two components show changes in size, as well as changes related to sagittal and metopic suture fusion. These are the two largest craniosynostosis classes in the dataset. The third principal component is associated with head asymmetry, resulting from non-synostotic positional plagiocephaly subjects in the control class. The pathology-specific submodels also depict the typical head deformities observed in clinical studies and are best used for controlled sampling of labeled synthetic instances.

From the tested template morphing methods, none of the approaches clearly outperformed the other ones. We considered N-ICP-A and iterative coherent point drift with Laplace–Beltrami regularized projection (ICPD-LBRP) to be the two most promising methods. Our decision to use N-ICP-A over ICPD-LBRP for the publicly available model was motivated by the smaller vertex-to-nearest-neighbor distances and the higher model compactness. Landmark errors are typically considered the gold standard, but as our model is concerned with craniosynostosis and the landmarks are located primarily on the face, we deemed them less important for this model.

A comparison with other craniosynostosis-related ssm is difficult as we propose the first publicly available model of infants. In the medical field, studies that used shape models [49,50] did not include quantitative metrics such as landmark error, compactness, generalization, and specificity. The most comparable ssm might be the Liverpool-York-Model [21,24], as it is a full-head model and also contains a submodel comprising children from 2 to 15 years of age. Compared with the Liverpool-York head model, our model is more compact and has a lower specificity error and lower vertex-to-nearest-neighbor distances, but a higher generalization and landmark error. However, a direct comparison may not be meaningful considering that both the dataset and the used mesh resolution are different. Our training data contain only children younger than 1.5 years, so the total variance of our model might be smaller by nature. However, by including a similar LBRP approach in our analysis, we show that our model performs comparable to state-of-the-art models.

The control class of our study was assembled by the scans of children who visited the hospital without indication to be treated surgically. This includes patients who were diagnosed being healthy and patients who were diagnosed having mild head deformities due to positional plagiocephaly. Thus, the control model represents a mixed group of children and should be used with caution when generating healthy subjects.

Using pca or WPCA assumes that the training data follow a multivariate normal distribution. This assumption does not hold up for a head model that includes different pathology classes. With respect to the classification, pca serves as a re-parameterization and ultimately as a dimensionality reduction procedure. This seems to be one of the key elements of our classification approach. pca is the de facto standard method for pdm generation, although some authors have proposed some alternatives for specific cases. probabilistic principal component analysis (PPCA) has been proposed for datasets with missing data or outliers [51], but has higher computational costs. As we have a regular mesh and removed corrupt scans before establishing dense correspondence, we employed WPCA.

Many authors proposed modifications to further improve ssm. With respect to template morphing, multiple correspondences can be taken into account [52]. Some possible improvements for the statistical modeling include the use of PPCA [51], reparameterization of training shapes [53], the use of part-based models [54], or combining multiple models [55,56]. Gaussian process morphable models (GPMMs) [57] model deformations as Gaussian processes, which increases model flexibility by the use of prior models, combining kernels, and operating in the continuous domain. Domain-specific Gaussian process shape deformations [20] can also be used for model building. These methods might further improve our model.

## 5. Conclusions

We presented a craniosynostosis classification pipeline using the parameter vector of an ssm. We achieved state-of-the-art results comparable to both ct data and photogrammetric scans, and to the best of our knowledge, we tested it on the largest craniosynostosis-specific dataset to date. We also presented the first full-head model of craniosynostosis patients and made it publicly available. We included pathology-specific submodels, ready-to-use sampled instances of each submodel, and a texture model. Our model performs similar to state-of-the-art head models with respect to morphing and model metrics and captures craniosynostosis-specific features. Finally, we showcased two original craniosynostosis-specific applications of our model.

## Figures and Tables

**Table 1:** Confusion matrix, sensitivity, and specificity for linear discriminant analysis (LDA), nonrigid iterative closest points affine (N-ICP-A) and the optimal number of components (44) using stratified 10-fold cross-validation. Con = control, Cor = coronal, Sag = sagittal, Met = metopic.

True Class	Predicted Class				Sensitivity	Specificity
	Con	Cor	Met	Sag		
Con	178	0	0	0	1.000	0.958
Cor	5	17	0	0	0.773	1.000
Met	0	0	56	0	1.000	1.000
Sag	3	0	0	108	0.973	1.000
G-mean					0.931	
Total accuracy						0.978

**Table 2:** Mean error and standard deviation for the N-ICP-A method.

Mean Landmark Error (mm)	Mean Vertex-to-Nearest-Neighbor Distance (mm)	Mean Surface Normals Deviations (Degree)
6.533±1.796	0.007±0.003	33.488±1.578

**Table 3:** Number of principal components included in the publicly available shape model data under Creative Commons license CC-BY-NC 4.0.

Model	Included Principal Components
Full shape model	100
Texture model	100
Control model	30
Sagittal model	30
Metopic model	25
Coronal model	15

## Data Availability

Patient data not available due to data protection laws. Program code available upon reasonable request. Publicly available shape model data available online at: https://doi.org/10.5281/zenodo.6390158 (accessed on 25 May 2022).

## Supplementary Materials

The following supporting information can be downloaded at: https://www.mdpi.com/article/10.3390/diagnostics12071516/s1, References [21,24,34,37,58,59] are cited in the supplementary materials.
