# Supplementary material for: A Radiation-Free Classification Pipeline for Craniosynostosis Using Statistical Shape Modeling
Source: Diagnostics (Basel). 2022 Jun 21;12(7):1516. doi: 10.3390/diagnostics12071516 (PMC9323148; doi:10.3390/diagnostics12071516)
Supplement: Supplementary file 1 [file diagnostics-12-01516-s001.zip › diagnostics-1765032-supplementary.pdf]

A radiation-free classification pipeline for craniosynostosis using statistical shape modeling

Supplementary Materials

S1 Landmarks

Table S1 shows the landmarks annotated by the medical staff.

Table S1: Landmarks on 3D surface scans provided by the medical staff. We use the cephalometric landmark notation of [1].

Landmark	Abbreviation
Tragion (left and right)	(t_{l}) and (t_{r})
Sellion	(se)
Exocanthion (left and right)	(ex_{l}) and (ex_{r})
Subnasale	(sn)
Labiale Superius	(ls)
Otobasion superius (left and right)	(obs_{l}) and (obs_{r})
Soft tissue gnathion	(gn)

S2 Description of morphing methods

S2.1 Description of optimal step nonrigid iterative closest points methods

To be consistent with the notation in the original paper [2] for the description of the optimal step nonrigid iterative closest points (OS-N-ICP) methods, we change notation. The n_{p} template points are expressed as $\mathbf{V} \in \mathbb{R}^{n_{\mathrm{p}} \times 3}$.

The unknown affine transformations are defined as $\mathbf{X} \in \mathbb{R}^{4n_{\mathrm{p}} \times 3}$. The full cost function can be expressed as $E(\mathbf{X}) = \alpha E_{\mathrm{s}}(\mathbf{X}) + E_{\mathrm{d}}(\mathbf{X}) + \beta E_{\mathrm{l}}(\mathbf{X})$. The stiffness term $E_{\mathrm{s}}(\mathbf{X})$ can be described as the Kronecker product $\otimes$ of the mesh topology matrix $\mathbf{M} \in \mathbb{R}^{n_{\mathrm{e}} \times n_{\mathrm{p}}}$ with n_{e} denoting the number of edges and n_{p} the number of points. The weight matrix $\mathbf{G} \in \mathbb{R}^{4\times4} = \mathrm{diag}(1, 1, 1, \gamma)$ between rotational and skew parts against translational parts [2]:

$$E_{\mathrm{s}}(\mathbf{X}) = \|(\mathbf{M} \otimes \mathbf{G})\mathbf{X}\|_F^2 . \quad \text{(S1)}$$

$\mathbf{M}$ describes the connections between neighboring vertices (we use the node-arc incidence matrix [3] in which for each edge r we set $\mathbf{M}(r,i) = -1$ and $\mathbf{M}(r,j) = 1$). The distance term $E_\mathrm{d}(\mathbf{X})$ describes how close the displaced template vertices are to the target vertices and can be written as:

$$E_\mathrm{d}(\mathbf{X}) = \|\mathbf{W}(\mathbf{D}\mathbf{X} - \mathbf{U})\|_F^2 . \tag{S2}$$

$\mathbf{W} \in \mathbb{R}^{n_\mathrm{P} \times n_\mathrm{P}}$ is a diagonal weighting matrix which allows assigning different weights to each transformation. The sparse displacement matrix $\mathbf{D} \in \mathbb{R}^{n_\mathrm{P} \times 4n_\mathrm{P}}$ is a diagnoal matrix with the homogeneous points $v_i = [x_i, y_i, z_i, 1]^\mathrm{T}$ as its diagnoal elements mapping the homogeneous template points to the respective affine transforms. $\mathbf{U} \in \mathbb{R}^{n_\mathrm{P} \times 3}$ denotes the found correspondences from the target points.

Finally, the landmark term $E_\mathrm{l}(\mathbf{X})$ is similar to the distance term while only the landmark points are considered:

$$E_\mathrm{l}(\mathbf{X}) = \|(\mathbf{D}_\mathrm{L}\mathbf{X} - \mathbf{U}_\mathrm{L})\|_F^2 . \tag{S3}$$

The complete cost function for nonrigid iterative closest points affine (N-ICP-A) can be written as:

$$E(\mathbf{X}) = \left\| \begin{bmatrix} \alpha\mathbf{M} \otimes \mathbf{G} \\ \mathbf{W}\mathbf{D} \\ \beta\mathbf{D}_\mathrm{L} \end{bmatrix} \mathbf{X} - \begin{bmatrix} \mathbf{0} \\ \mathbf{W}\mathbf{U} \\ \mathbf{U}_\mathrm{L} \end{bmatrix} \right\|_F^2 \tag{S4}$$

For the translation-only variant nonrigid iterative closest point translation (N-ICP-T), the unknown transformations are defined as translations $\mathbf{X} \in \mathbb{R}^{n_\mathrm{P} \times 3}$. The cost function is changed accordingly:

$$E(\mathbf{X}) = \left\| \begin{bmatrix} \alpha\mathbf{M} \\ \mathbf{W}\mathbf{I}_{n_\mathrm{P}} \end{bmatrix} \mathbf{X} - \begin{bmatrix} \mathbf{0} \\ \mathbf{W}(\mathbf{U} - \mathbf{V}) \end{bmatrix} \right\|_F^2 \tag{S5}$$

S2.2 Description of Laplace-Beltrami regularized projection methods

Laplace-Beltrami regularized projection (LBRP) [4, 5] relies on mutual correspondences between template and target and uses the Laplace-Beltrami (LB) operator $\mathbf{L}_0 \in \mathbb{R}^{n_\mathrm{P} \times n_\mathrm{P}}$ computed on the original template as a regularization, controlled by the stiffness parameter λ. A higher λ puts more weight to the LB term of the equation, leading to a mesh which retains its original shape. For a low λ, the original template shape is disregarded and is mapped closer to the target mesh, which might lead to irregularities in the projection. This template projection step can be described using [4, 5]:

$$\begin{bmatrix} \lambda\mathbf{L}_0 \\ \mathbf{S}_\mathrm{X} \end{bmatrix} \mathbf{X} = \begin{bmatrix} \lambda\mathbf{L}_0\mathbf{X}_0 \\ \mathbf{S}_\mathrm{Y}\mathbf{Y} \end{bmatrix}, \tag{S6}$$

The two Boolean selection matrices $\mathbf{S}_\mathrm{X} \in [0,1]^{k \times n_\mathrm{n}}$ and $\mathbf{S}_\mathrm{Y} \in [0,1]^{k \times n_\mathrm{t}}$ select the k correspondences on the template and target. n_p denotes the number of template points, n_t the number of target points.

For the two-step Laplace-Beltrami regularized projection, we essentially perform this template projection twice, first to adapt the template to the target and then refining it with decreased

stiffness to let it deform more strongly to the target. This is a similar approach to the template adaption described in [5].

For the iterative coherent point drift with Laplace-Beltrami regularized projection [5], we first employ the adaptive template projection with a high λ, then alternate between the rigid coherent point drift (CPD) and nonrigid CPD [6] and conclude with the adaptive template projection with a small λ. For our dataset, this increased robustness compared to using the affine CPD variant as proposed in [5]. For further reading on the iterative coherent point drift and the template adaption of the LBRP, the reader is referred to [5].

S2.3 Hyperparameters for template morphing

Table S2 lists the hyperparameters used in each method.

Table S2: Hyperparameters used for the template morphing approaches.

Two-step Laplace-Beltrami regularized projection (2S-LBRP) (notation of [5])	
Stiffness first morph	$\lambda_1 = 10$
Stiffness second morph	$\lambda_2 = 0.1$
Iterative coherent point drift with Laplace-Beltrami regularized projection (ICPD-LBRP) (notation of [5])	
Stiffness first morph	$\lambda_1 = 10$
Iterative coherent point drift (ICPD)-Loop	For each iteration, perform first *cpdRigid*, then *cpdNonrigid*
cpdNonrigid smoothing weight:	3
cpdNonrigid tolerance	$1 \cdot 10^{-5}$
Exit condition	fewer than 1 % of nearest neighbors between iterations change
Stiffness second morph	$\lambda_2 = 0.1$ with Laplace matrix resulting from first morph
Nonrigid iterative closest points affine (N-ICP-A) and nonrigid iterative closest point translation (N-ICP-T) (notation of [2])	
Iterations	$n = 80$
Stiffness parameter α in iteration n	$\alpha_n = 10^8 \cdot 0.8^n$
Landmark weight in iteration n β	if $n < 51$ $\beta_n = 1$, else $\beta_n = 0$
Exit condition ϵ for each fixed stiffness α	$\epsilon < 100$
Valid normals for correspondence establishment φ	$\varphi < 45°$
Rotation weight γ	$\gamma = 1$

S3 Evaluation of the morphing methods

S3.1 Morphing evaluation

We present mean and standard deviations for each error metric in Table S3. Cumulative errors for each metric show the distribution of each error and are displayed in Figure S1. LBRP methods showed smaller landmark errors and larger surface normal deviations compared to OS-N-ICP. N-ICP-A had the lowest vertex-to-nearest-neighbor distance errors. Surface normal deviations were for all methods larger than 17°.

Table S3: Mean error and standard deviation for each morphing method. Boldface shows smallest error for each metric.

Morphing method	Mean landmark error (mm)	Mean vertex-to-nearest-neighbor distance (mm)	Mean surface normals deviations (degree)
Nonrigid iterative closest points affine (N-ICP-A)	6.533 ± 1.796	$\mathbf{0.007 \pm 0.003}$	33.488 ± 1.578
Nonrigid iterative closest point translation (N-ICP-T)	5.699 ± 1.789	0.302 ± 0.01	23.242 ± 1.849
Two-step Laplace-Beltrami regularized projection (2S-LBRP)	4.185 ± 1.205	0.785 ± 0.17	$\mathbf{20.392 \pm 1.466}$
Iterative coherent point drift with Laplace-Beltrami regularized projection (ICPD-LBRP)	$\mathbf{4.071 \pm 1.163}$	0.272 ± 0.049	29.255 ± 2.12

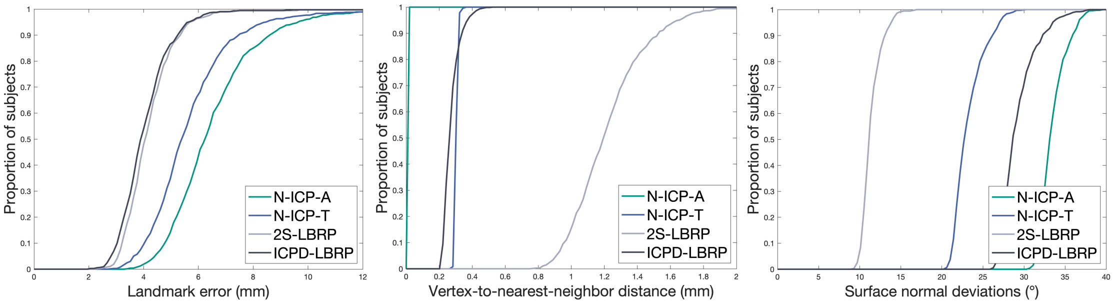

Figure S1: Proportion of subjects with mean landmark error, vertex-to-nearest-neighbor-distances, and surface normal. We compared nonrigid iterative closest points affine, nonrigid iterative closest point translation, two-step Laplace-Beltrami regularized projection (2S-LBRP), and iterative coherent point drift with Laplace-Beltrami regularized projection (ICPD-LBRP). The graph shows the proportion of subjects less than the abscissa value. Higher is better.

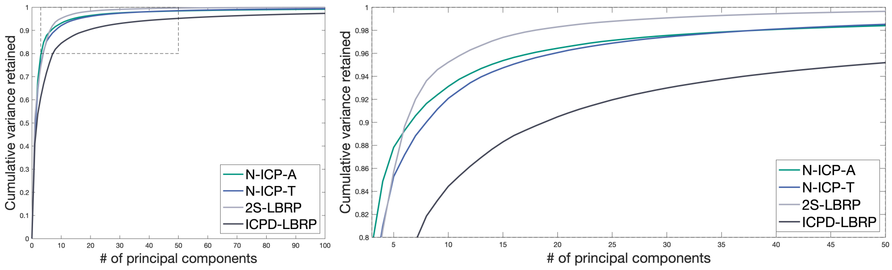

Figure S2: Compactness as a function of the number of principal components of the full shape model. We compared nonrigid iterative closest points affine (N-ICP-A), nonrigid iterative closest point translation (N-ICP-T), two-step Laplace-Beltrami regularized projection (2S-LBRP), and iterative coherent point drift with Laplace-Beltrami regularized projection (ICPD-LBRP). Left: full compactness, right: zoom-in. A higher value is better.

Figure S2 shows the compactness of the statistical shape model. The most compact models were produced by N-ICP-A and N-ICP-T, while generalization error and specificity error were larger (Figure S3).

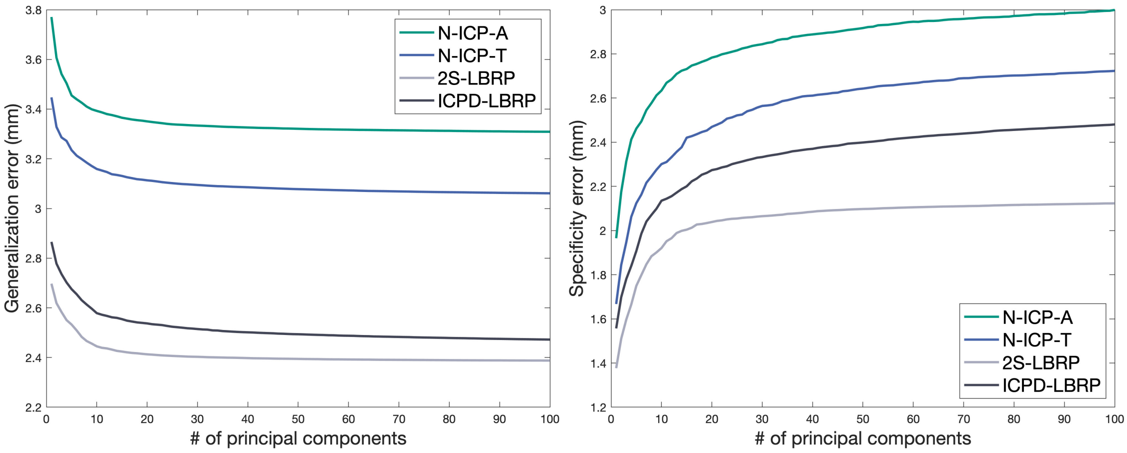

Figure S3: Generalization and specificity errors as functions of the number of principal components of the full shape model. We compared nonrigid iterative closest points affine (N-ICP-A), nonrigid iterative closest point translation (N-ICP-T), two-step Laplace-Beltrami regularized projection (2S-LBRP), and iterative coherent point drift with Laplace-Beltrami regularized projection (ICPD-LBRP). Left: generalization error, right: specificity error. For both metrics, a lower error is better.

S3.2 Classfication evaluation

In Table S4 we show the classification results per morphing method and classification approach. Linear discriminant analysis (LDA) consistently yielded the highest accuracy regardless of the morphing method. Using LDA, all morphing methods obtained accuracies of 97.0 % or higher.

Note that N-ICP-T model scores the highest accuracy although it did not perform best in any of the evaluation criteria.

Table S4: Highest accuracy for each classifier and morphing methods. Optimal number of principal components is given in brackets. Underlined accuracy indicates optimal classifier per method and boldface optimal classifier overall.

Morphing method	LDA	support vector machine	naïve Bayes	k-nearest-neighbors	bagged decision tree
Nonrigid iterative closest points affine	0.978 (44)	0.962 (46)	0.946 (23)	0.916 (23)	0.826 (22)
Nonrigid iterative closest point translation	**0.981** (54)	0.959 (23)	0.946 (28)	0.910 (9)	0.842 (10)
Two-step Laplace-Beltrami regularized projection	0.970 (50)	0.970 (31)	0.959 (23)	0.946 (10)	0.861 (8)
Iterative coherent point drift with Laplace-Beltrami regularized projection	0.975 (91)	0.962 (13)	0.951 (23)	0.951 (10)	0.847 (8)

References

[1] Gwen R.J. Swennen, Filip Schutyser, and Jarg-Erich Hausamen. *Three-Dimensional Cephalometry.* Springer Berlin Heidelberg, Berlin, Heidelberg, 2006.

[2] Brian Amberg, Sami Romdhani, and Thomas Vetter. Optimal Step Nonrigid ICP Algorithms for Surface Registration. In *2007 IEEE Conference on Computer Vision and Pattern Recognition*, pages 1–8, Minneapolis, MN, USA, June 2007. IEEE.

[3] Melvyn W. Jeter. *Mathematical Programming: An Introduction to Optimization.* Number 102 in Monographs and Textbooks in Pure and Applied Mathematics. M. Dekker, New York, 1986.

[4] Hang Dai, Nick Pears, and Christian Duncan. A 2D Morphable Model of Craniofacial Profile and Its Application to Craniosynostosis. In María Valdés Hernández and Víctor González-Castro, editors, *Medical Image Understanding and Analysis*, volume 723, pages 731–742. Springer International Publishing, Cham, 2017.

[5] Hang Dai, Nick Pears, William Smith, and Christian Duncan. Statistical Modeling of Craniofacial Shape and Texture. *International Journal of Computer Vision*, 128(2):547–571, February 2020.

[6] Andriy Myronenko and Xubo Song. Point Set Registration: Coherent Point Drift. *IEEE Transactions on Pattern Analysis and Machine Intelligence*, 32(12):2262–2275, December 2010.
